# Modulatory effect of *Canavalia gladiata* lectin on quinolone activity against multidrug-resistant and efflux pump-positive *Staphylococcus aureus*

**DOI:** 10.3389/fphar.2026.1953977

**Published:** 2026-10-29

**Authors:** Dárcio Luiz de Sousa Júnior, Maria do Socorro Costa, Sheila Alves Gonçalves, Cicera Laura Roque Paulo, Isaac Moura Araújo, Ana Cecília Calixto Donelardy, Antonia Railene de Souza Rodrigues, Rafael Barbosa de Moura, Thiago Sampaio de Freitas, Janaína Esmeraldo Rocha, Henrique Douglas Melo Coutinho, Francisco Nascimento Pereira Júnior

**Affiliations:** 1 Federal University of Cariri, Brejo Seco, Ceará, Brazil; 2 Regional University of Cariri, Crato, Ceará, Brazil

**Keywords:** antibiotic adjuvant, antimicrobial activity, canavalia gladiata, efflux pump, mate, MDR, multidrug-resistant bacteria, plant lectins

## Abstract

Clinically relevant bacteria have increasingly developed resistance to antimicrobial agents, reducing the effectiveness of conventional antibiotic therapies. In this context, antibiotic adjuvants capable of restoring or enhancing the activity of existing drugs represent a potential strategy against multidrug-resistant bacteria. This study evaluated the potential of a lectin isolated from *Canavalia gladiata* seeds (CGL) as an antibiotic-modulating agent against multidrug-resistant and efflux pump-positive bacterial strains. CGL was purified by affinity chromatography on Sephadex G-50, and its direct antibacterial activity was evaluated by determining the minimum inhibitory concentration (MIC). Its modulatory effects were subsequently assessed in combination with conventional antibiotics and in assays involving strains carrying the NorA and MepA efflux pumps. CGL showed weak intrinsic antibacterial activity against all tested strains, with MIC values ≥1,024 μg/mL. However, when used at a sub-inhibitory concentration, CGL moderately enhanced the activity of norfloxacin against *Staphylococcus aureus* 10, reducing the MIC from 256 to 170.6 μg/mL, and ciprofloxacin against *S. aureus* K2068, reducing the MIC from 32 to 13.3 μg/mL. These effects did not restore bacterial susceptibility to clinically established therapeutic breakpoints. In contrast, no significant enhancement was observed for the other antibiotic combinations, and some associations resulted in increased MIC values. Therefore, CGL does not appear to act as a direct antibacterial agent under the conditions evaluated, but may have potential as an antibiotic adjuvant, particularly in combination with quinolones against selected *S. aureus* strains. Further mechanistic, toxicity, and *in vivo* studies are required to determine its applicability as an antibiotic-modulating agent.

## 1 Introduction

Antimicrobial resistance (AMR) remains one of the major challenges to public health, directly impacting the treatment of bacterial infections. This phenomenon affects populations across all social levels and in different regions of the world. In this context, the World Health Organization (WHO) has adopted important measures for control, surveillance, and monitoring, since the inappropriate use of antibiotics is recognized as the main factor driving the advancement of resistance (World Health Organization, 2024).

Drugs routinely used with a broad spectrum of activity against bacteria are the main medications affected by AMR, leading physicians and specialists to employ combinations of antibiotics, often associated with increased adverse effects. This not only reduces the therapeutic arsenal but also further decreases the patient’s chances of cure (Silva A. R. P. et al., 2024).

In this scenario, both Gram-negative and Gram-positive bacteria have already been reported with AMR. *Escherichia coli* strains currently display a highly diversified resistance profile; this microorganism mainly causes infections in the gastrointestinal and urinary tracts but has also been associated with more severe diseases (Costa A. R. et al., 2024). *Staphylococcus aureus* is another medically relevant bacterium that has been exhibiting AMR; although it is part of the normal human microbiota, inhabiting the skin and mucous membranes, it can develop into high-grade infections. These examples acquire resistance through several mechanisms, such as enzymes that degrade or inactivate the drug and proteins like efflux pumps (Silva J. C. et al., 2024; Araújo et al., 2025).

In light of the shortage of new antimicrobial agents and the rapid and growing resistance to them, the academic community and scientists have been seeking alternatives to improve this scenario. The search for new compounds, especially of natural origin, represents the most promising path, given their extensively elucidated biological activities. These compounds not only show direct action against microorganisms but also demonstrate the potential to modify the effects of previously ineffective drugs (Silva L. et al., 2024).

From this perspective, lectins proteins capable of binding to carbohydrates have been studied in various fields, displaying, among many biological activities, antibacterial, antifungal, antiprotozoal, antitumor, and immunomodulatory effects, either directly or indirectly, due to their interaction with macromolecules (Santos et al., 2021; Santos et al., 2023; Fonseca et al., 2022a; Fonseca et al., 2022b; Costa M. S. et al., 2024).

Therefore, considering the impact of these infections on public health and the need for new antimicrobial substances, the present study aimed to evaluate the activity of a lectin extracted from *Canavalia gladiata* seeds against standard, multidrug-resistant, and efflux pump carrying strains. Given that other members of the *Canavalia genus*, such as *C. ensiformis* (ConA), exhibit anti-biofilm and antibacterial properties, the investigation of CGL as an antibiotic adjuvant represents an important step in exploring the biological versatility of this protein family.

## 2 Materials and methods

### 2.1 Biological material and protein extraction

*Canavalia gladiata* seeds were provided by the Laboratory of Biologically Active Molecules of the Federal University of Ceará. The seeds were commercially obtained, and a voucher specimen of the species was deposited and identified at the Prisco Bezerra Herbarium–UFC under the code EAC0032586.

The seeds of *C. gladiata* were ground into a fine flour using an electric coffee grinder. Soluble proteins were extracted in 0.15 M NaCl at a ratio of 1:10 (w/v), under constant stirring for 4 h at room temperature. After homogenization, the extract was centrifuged at 4,000 × g at 4 °C for 10 min, and the supernatant was collected and filtered through filter paper, which was then used in the subsequent chromatography steps.

### 2.2 Sephadex G-50 gel affinity chromatography

The crude extract was applied to a Sephadex® G-50 affinity column previously equilibrated with 0.15 M NaCl solution. Eight hours after the material was loaded onto the column, the unbound fraction was washed with the same solution, and the *C. gladiata* lectin (CGL) was eluted with 0.1 M D-glucose in 0.15 M NaCl. The eluted fractions were monitored spectrophotometrically at 280 nm absorbance. The collected material was dialyzed against distilled water for 9 h, with water changes every hour. The fraction containing CGL was then lyophilized, and the purification profile was evaluated by SDS-PAGE (Laemmli, 1970).

The purification of CGL followed a standardized protocol previously validated through X-ray diffraction and biochemical characterization (Moreno et al., 2004; Delatorre et al., 2007). The identity and consistency of the isolated fraction were confirmed by SDS-PAGE, using the established 24 kDa monomeric profile as a quality control benchmark. While the final mass of the lyophilized lectin was recorded (0.139 g), specific purification metrics such as recovery yield and purification factor were not calculated for this particular batch, as the primary objective was the evaluation of biological activity.

### 2.3 Microorganisms, culture media, and substances

For the microbiological assays, six bacterial strains were used: two reference strains, *Escherichia coli* ATCC 10536 and *Staphylococcus aureus* ATCC 25923; two multidrug-resistant clinical isolates, *E. coli* 06 (urine isolate) and *S. aureus* 10 (rectal swab isolate); and two efflux pump–carrying strains, *S. aureus* 1199B (NorA-positive) and *S. aureus* K2068 (MepA-positive). The strains were provided by the Laboratory of Microbiology and Molecular Biology (LMBM-URCA). Their resistance profiles are described in Table 1. All microbiological assays were performed in technical triplicate.

**TABLE 1:** Origin of strains and bacterial resistance profile.

Bacterial strains	Origin	Resistance profile
*Staphylococcus aureus* 10	Rectal swab	Amc, amox, amp, asb, azi, cefa, cef, cf, cip, cla, clin, ery, lev, mox, oxa, pen
*Escherichia coli* 06	Urine	Asb, cefa, cef, cfo, cpm, ctx
*Staphylococcus aureus* 1199B	LMBM	Expresses the NorA efflux pump, resistant to norfloxacin
*Staphylococcus aureus* K2068	LMBM	Expresses the MepA efflux pump, resistant to ciprofloxacin

Amc – Amoxicillin + clavulanic acid, Amox – Amoxicillin, Amp – Ampicillin, Asb – Ampicillin + Sulbactam, Azi – Azithromycin, Cefa – Cefadroxil, Cef – Cephalexin, Cfo – Cefoxitin, Cip – Ciprofloxacin, Cla – Clarithromycin, Clin – Clindamycin, Cpm – Cefepime, Ctx – Ceftriaxone, Ery – Erythromycin, Lev – Levofloxacin, Mox – Moxifloxacin, Oxa – Oxacillin, Pen – Penicillin. LMBM: laboratory of microbiology and molecular biology.

Chlorpromazine (CPZ), m-chlorophenylhydrazone carbonyl cyanide (CCCP), and ethidium bromide were obtained from Sigma Aldrich Co. Ltd. and dissolved in sterile distilled water, protected from light. CCCP was dissolved in a 1:1 methanol/water solution. The drugs used included the antibiotics ampicillin, ciprofloxacin, gentamicin, and norfloxacin, while the test substance was the lectin from *C. gladiata* seeds (CGL). Except for CGL and gentamicin, which were dissolved in sterile water, all other compounds were initially dissolved in dimethyl sulfoxide (DMSO) and subsequently diluted in sterile water to reach a final concentration of 1,024 μg/mL. In all assays, the final concentration of DMSO or methanol in the wells did not exceed 1% (v/v) to ensure no interference with bacterial growth (Oluyele, 2025).

The culture media, obtained from Acumedia Manufacturers Inc., were Heart Infusion Agar (HIA) for strain revival, prepared according to the manufacturer’s instructions, and Brain Heart Infusion (BHI) at a 10% concentration for the microdilution assays.

### 2.4 Determination of minimum inhibitory concentration (MIC)

The MIC was determined by broth microdilution with adaptations. Initially, the strains were streaked from stock onto HIA medium, and after 24 h, colonies of the replicated cells were collected and inoculated into test tubes containing 3 mL of sterile saline solution, which were then compared to a 0.5 McFarland standard (∼1.5 × 10^8^ CFU/mL). After standardization, microtubes containing 900 μL of 10% BHI medium and 100 μL of the inoculum were prepared, totaling 1.0 mL.

For this assay, 96-well plates (horizontal orientation) were used. In each well, 100 μL of the microtube contents were added, followed by 100 μL of CGL, which was serially diluted, with concentrations ranging from 512 μg/mL to 8 μg/mL up to the penultimate well; the last well served as the growth control. All tests were performed in technical triplicate, and plates were incubated for 24 h at 37 °C. To determine bacterial MIC, 20 μL of resazurin (7-hydroxy-3H-phenoxazin-3-one 10-oxide) was added. Readings were based on the color change caused by resazurin: a shift from blue to pink indicates bacterial growth, while the persistence of blue indicates inhibition. The MIC is defined as the lowest concentration at which no bacterial growth is observed (CLSI, 2015). Appropriate controls were included in each plate: sterility control (BHI only), growth control (BHI + inoculum), and vehicle control (BHI + DMSO/methanol + inoculum).

### 2.5 Analysis of the combined activity and modulating potential of antibiotics

To assess the indirect effect of CGL on antibiotic activity, a modulation assay was performed (Coutinho et al., 2008). All bacterial inoculum preparations followed the procedure described in the previous section. Brain Heart Infusion (BHI) at a 10% concentration was selected as the growth medium to ensure the viability of the clinical isolates while preventing high nutrient concentrations from masking the modulatory activity of CGL or interfering with its binding capacity. After standardization, 150 μL of the inoculum and CGL at a sub-inhibitory concentration (MIC/8) were added to microtubes, and the total volume was adjusted to 1.5 mL.

A subinhibitory concentration corresponding to MIC/8 was used because concentrations below the inhibitory threshold allow the evaluation of antibiotic-modulating effects without substantial interference from the intrinsic antimicrobial activity of the tested substance. This approach is widely employed in studies investigating resistance modulation and efflux pump inhibition by natural products and bioactive compounds (Silva J. C. et al., 2024).

Again, 96-well plates were used, arranged vertically. In each well, 100 μL of the microtube contents were added, followed by 100 μL of the antibiotic, which was serially diluted, with concentrations ranging from 512 μg/mL to 0.5 μg/mL up to the penultimate well; the last well served as the growth control. The experiment was performed in technical triplicate, and plates were incubated for 24 h at 37 °C. To determine bacterial MIC, 20 μL of resazurin was added, showing the same color change indicative of bacterial growth or inhibition as in the previous assay.

### 2.6 Evaluation of NorA and MepA efflux pump inhibition

To evaluate whether the test substance acts as a potential modulator of resistance in efflux-positive strains, a comparative study with standard inhibitors was performed, a comparative study with standard inhibitors was performed, analyzing the ability of both to reduce the minimum inhibitory concentration (MIC) of ethidium bromide (Silva J. C. et al., 2024). The standard inhibitors CCCP and chlorpromazine were used to validate the expression of efflux pumps by the tested strain. Efflux-related modulation was assessed using a sub-inhibitory concentration (MIC/8), of the inhibitors and the evaluated CGL. The inclusion of these controls was essential to validate the efflux-mediated resistance phenotype of the *S. aureus* 1199B (NorA) and K2068 (MepA) strains. CCCP and chlorpromazine were included as reference compounds commonly used in studies investigating efflux-related mechanisms. CCCP dissipates the proton motive force (PMF), which is required for the activity of many energy-dependent transport systems, whereas chlorpromazine has been reported to affect bacterial efflux-associated processes. However, because the assays were based on bacterial growth after 24 h of incubation, changes in MIC values in the presence of these compounds cannot be attributed exclusively to efflux inhibition. In particular, the effects of CCCP may also reflect broader physiological disturbances associated with PMF dissipation. Therefore, these compounds were used as pharmacological reference agents, but their effects were not considered direct evidence of NorA or MepA pump activity or inhibition.

Bacterial suspensions were initially adjusted to the 0.5 McFarland standard (∼1.5 × 10^8^ CFU/mL) and subsequently diluted in BHI broth to obtain the final inoculum concentration recommended for microdilution assays, Consequently, 150 μL of each bacterial inoculum suspended in saline solution was added, along with the inhibitors and CGL (MIC/8), and the volume was made up with BHI. The mixtures were then transferred to 96-well microdilution plates, to which 100 μL of ethidium bromide and antibiotics were added in serial 1:1 dilutions, ranging from 512 to 0.5. Plates were incubated at 37 °C for 24 h, and bacterial growth was evaluated using resazurin, as in the previous assays.

### 2.7 Statistical analysis

The central values and standard deviations were obtained according to the methodology of Freitas et al. (2021) for microbiological analysis using microdilution plates. For the tests with efflux pump-carrying bacteria, the data were analyzed using GraphPad Prism 9.0 software via a one-way ANOVA, and for analyses involving multidrug-resistant bacteria, a two-way ANOVA was used, both followed by a Bonferroni *post hoc* test, with a p-value <0.05 considered significant and a p-value >0.05 considered non-significant.

## 3 Results

After lyophilization, 0.139 g of lyophilized lectin was obtained. The purification of CGL is confirmed in Figure 1, which shows the SDS-PAGE of CGL, displaying a single band across the different concentrations analyzed, with a molecular weight determined to be 24 kDa.

**FIGURE 1:**
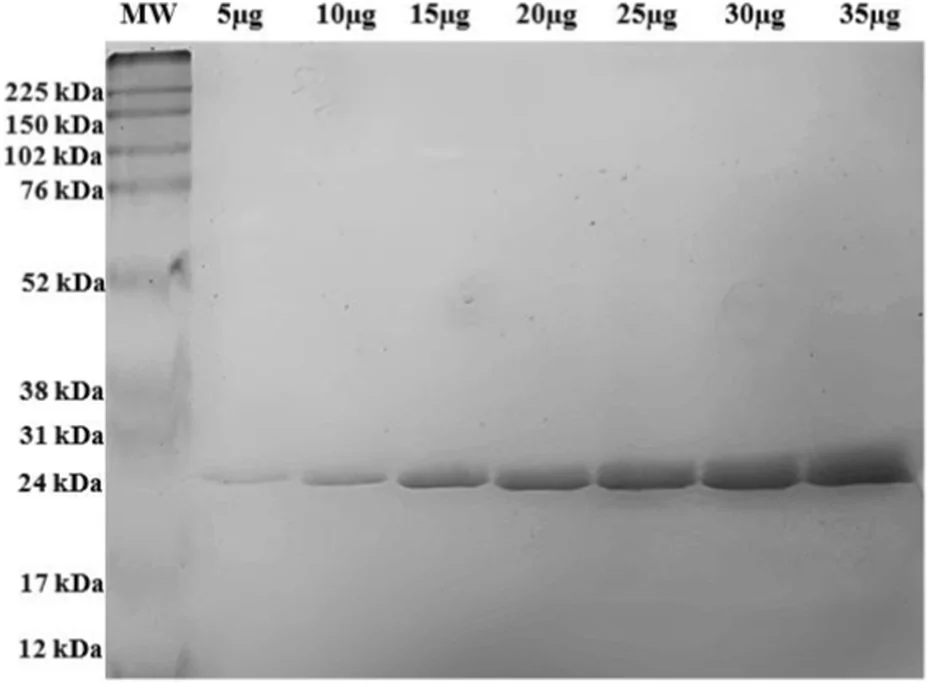
SDS-PAGE analysis of purified Canavalia gladiata lectin (CGL) obtained after affinity chromatography on Sephadex G-50, followed by dialysis and lyophilization. MW: molecular weight marker. Lanes containing 5, 10, 15, 20, 25, 30, and 35 µg of purified lectin showed a predominant band with an apparent molecular weight of approximately 24 kDa, consistent with previous reports for CGL.

The minimum inhibitory concentration (MIC) results of CGL against all analyzed bacteria (*S. aureus* 10, *S. aureus* ATCC 25923, *E. coli* 06, *E. coli* ATCC 10536, *S. aureus* 1199B, and *S. aureus* K2068) were not considered significant, presenting an MIC ≥1,024 μg/mL (Houghton et al., 2007). The data presented below (Figure 2) show the effect of combining CGL with the antibiotics norfloxacin, gentamicin, and ampicillin against the multidrug-resistant bacteria.

**FIGURE 2:**
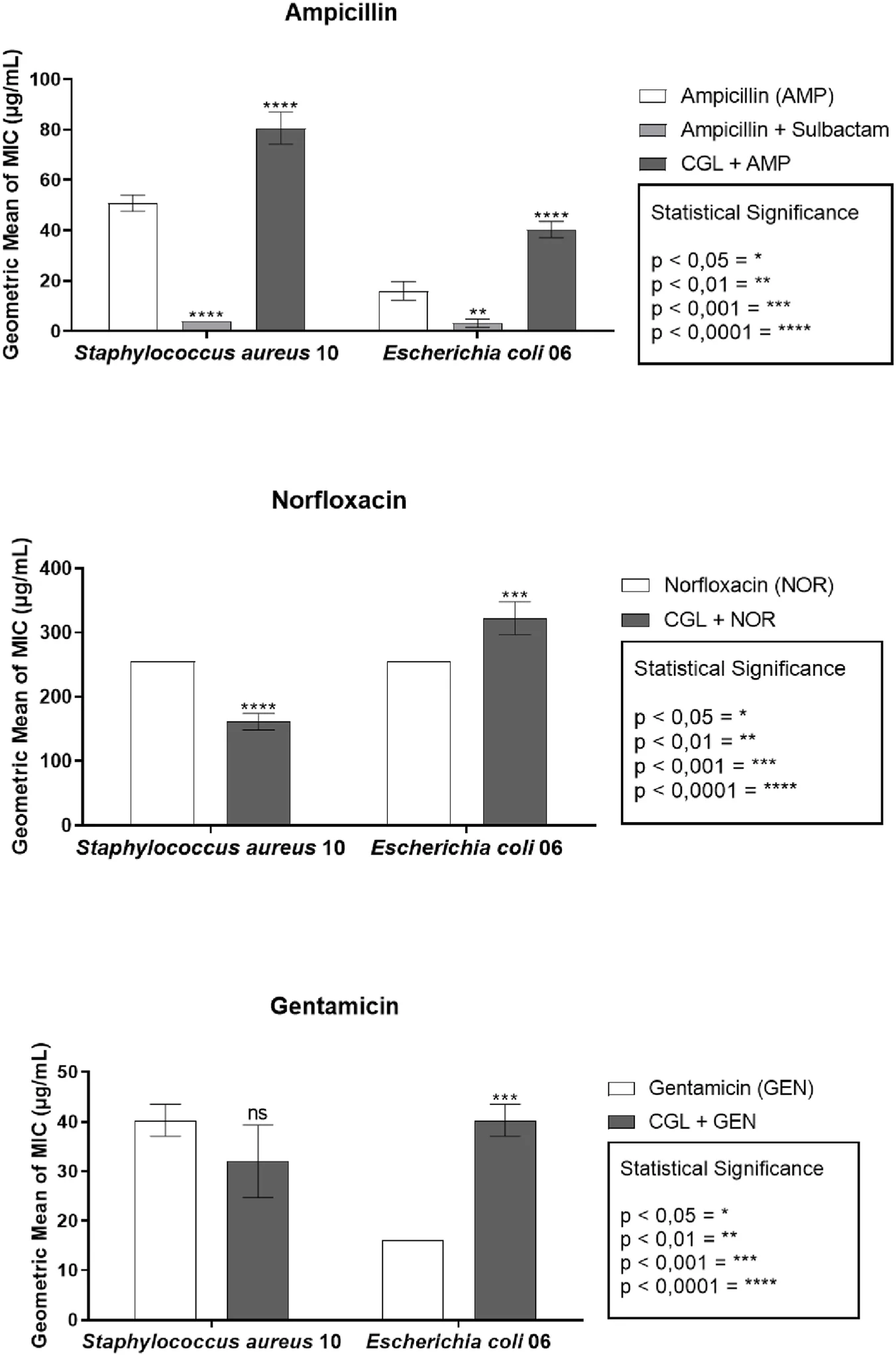
Associations of CGL with the antibiotics norfloxacin, ampicillin and gentamicin. Data are presented as mean ± standard deviation, with measurements taken in technical triplicate. Statistical analysis was performed using one-way ANOVA, followed by Bonferroni *post hoc* test. *p < 0.05, **p < 0.01, ***p < 0.001, ****p < 0.0001.

Of the three assays conducted, only the combination of CGL and norfloxacin against *S. aureus* 10 was considered significant, reducing the antibiotic concentration from 256 to 170.6 μg/mL, which suggests a modest modulatory effect on the antibiotic’s activity, though it was considered statistically significant (p < 0.0001 = ****). In all other cases, CGL further reduced the antibiotics’ efficacy, requiring a higher concentration to achieve the same effect. Detailed statistical results for the tested combinations are presented in the Supplementary Material.

In Figure 2, the combination tests with ampicillin did not show a significant effect of CGL; a considerable increase in the MIC was observed with this combination. However, it was also observed that, when combining ampicillin with sulbactam, both *S. aureus* 10 and *E. coli* 06 appear to exhibit, hypothetically, a possible β-lactamase mechanism; however, this remains purely speculative, since no confirmatory enzymatic or molecular assays were performed.

Figures 3, 4 present the results of the efflux pump inhibition assays and the combined effect of CGL, using *S. aureus* strains carrying the MepA and NorA pumps. In both *S. aureus* K2068 and 1199B, CGL failed to reduce the MIC of ethidium bromide and, in fact, caused a significant increase in its values (from 64 to 96 μg/mL and 512 μg/mL, respectively). This observed antagonism suggests that CGL does not act as a direct inhibitor of the MepA or NorA efflux pumps under the tested conditions. Instead, the lectin appears to interfere with the dynamics of these substrates in a manner that does not involve pump blockade.

**FIGURE 3:**
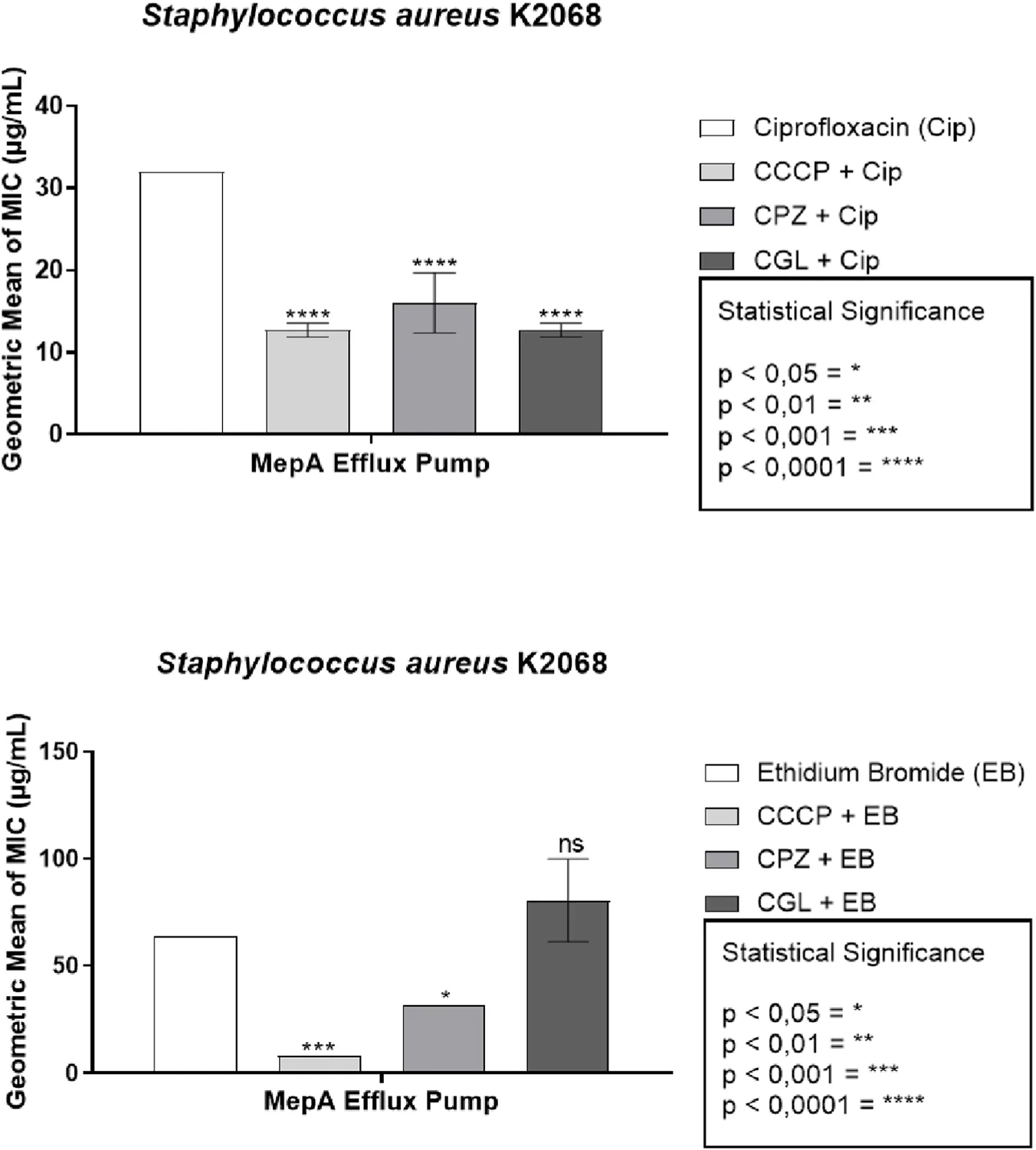
Inhibition assays of the MepA efflux pump, present in *S. aureus* K2068, from the association of CGL with antibiotic and ethidium bromide. Data are presented as mean ± standard deviation, with measurements taken in technical triplicate. Statistical analysis was performed using one-way ANOVA, followed by Bonferroni *post hoc* test. *p < 0.05, **p < 0.01, ***p < 0.001, ****p < 0.0001.

**FIGURE 4:**
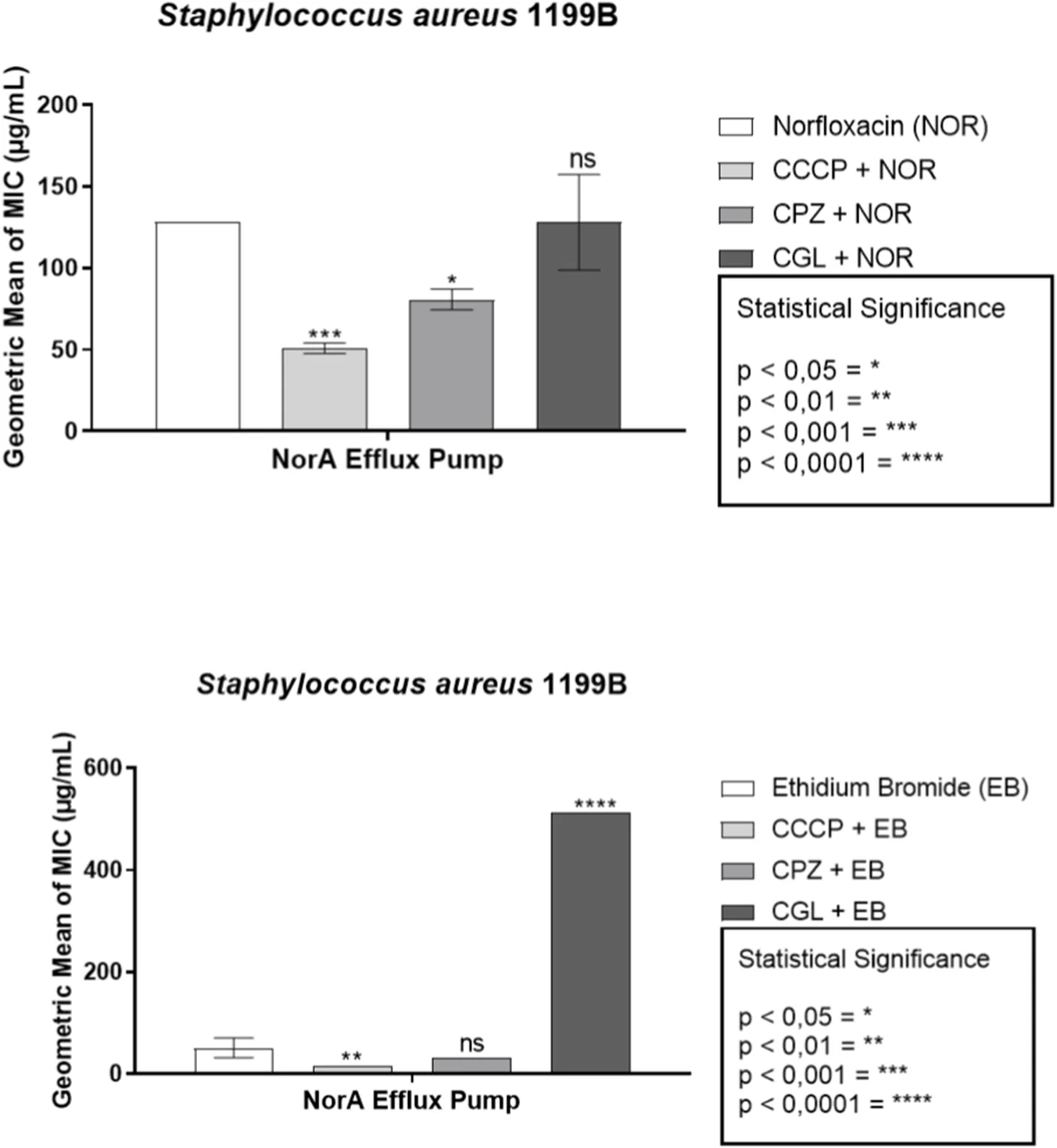
Inhibition assays of the NorA efflux pump, present in *S. aureus* 1199B, from the association of CGL with antibiotic and ethidium bromide. Data are presented as mean ± standard deviation, with measurements taken in technical triplicate. Statistical analysis was performed using one-way ANOVA, followed by Bonferroni *post hoc* test. *p < 0.05, **p < 0.01, ***p < 0.001, ****p < 0.0001.

The results of the CGL combination assay, both with the antibiotic and ethidium bromide, in the 1199B strain were considered not significant. In this case, CGL caused a possible antagonism when associated with norfloxacin, increasing its MIC from 128 to 149.3 μg/mL, and when combined with ethidium bromide, CGL also increased the MIC from 64 to 512 μg/mL.

## 4 Discussion

Prokaryotic organisms such as bacteria can possess intrinsic resistance to antibiotic drugs; that is, mutations enable them to expand their arsenal against these agents. Throughout their lifespan, these microorganisms can acquire various other resistance mechanisms, such as the synthesis of efflux pumps and enzymes that inactivate antibiotics, including β-lactamases. All these factors can increase the survival chances of many clinically relevant species (Matamoros-Recio et al., 2021; Li et al., 2023).

Lectins exert their antibacterial activity through multiple mechanisms, with the agglutination of cells or macromolecules being one of the most relevant. This effect results from their ability to specifically bind to carbohydrates present in biological membranes, promoting interactions that compromise microbial cell integrity. Studies by Tamilarasan et al. (2021) and Arokiyaraj et al. (2022) have demonstrated that lectins are capable of agglutinating both Gram-positive and Gram-negative bacteria, in addition to exhibiting significant minimum inhibitory concentrations (MICs), highlighting their potential as antimicrobial agents.

The SDS-PAGE profile revealed a predominant protein band with an apparent molecular mass of approximately 24 kDa, consistent with previous reports for CGL. Although this result indicates substantial enrichment of the lectin fraction, electrophoretic analysis alone is insufficient to confirm complete protein homogeneity (Moreno et al., 2004; Delatorre et al., 2007; Bezerra et al., 2007).

Directly, CGL did not exhibit significant activity, presenting an MIC of ≥1,024 μg/mL. The absence of direct antibacterial activity observed for CGL (MIC ≥1,024 μg/mL) indicates that, under the conditions evaluated, the lectin does not exhibit clinically relevant intrinsic antibacterial effects against the tested strains. According to commonly adopted criteria for natural products, MIC values above 1,000 μg/mL are generally considered indicative of weak or absent antimicrobial activity (Houghton et al., 2007). Therefore, the potential application of CGL may be more closely related to its modulatory effects when combined with conventional antibiotics than to a direct antibacterial action (Araújo et al., 2023).

In contrast, lectins from other legume species have previously demonstrated antibacterial effects against both Gram-positive and Gram-negative bacteria, which have been associated in the literature with their carbohydrate-binding properties and their ability to interact with microbial surfaces (El-Araby et al., 2020; Silva and Araújo, 2021; Jain et al., 2022). Although the present study did not investigate the mechanisms underlying the observed modulatory effects, these previous findings suggest possible pathways through which lectins may influence bacterial susceptibility.

In the present study, CGL showed a modulatory effect when combined with quinolone-class antibiotics. The lectin reduced the MIC of norfloxacin against *S. aureus* 10 and ciprofloxacin against *S. aureus* K2068; however, these reductions were moderate and did not restore bacterial susceptibility within clinically established therapeutic breakpoints. Therefore, although statistically significant, the biological and clinical relevance of these effects should be interpreted with caution.

Nevertheless, even modest reductions in MIC values may be relevant in studies involving natural products as antibiotic adjuvants, particularly against multidrug-resistant bacteria (Kumar et al., 2023; Zhai et al., 2023). These findings suggest a possible interaction between CGL and bacterial resistance mechanisms, including efflux systems, although the exact mechanism of action remains unclear. Thus, the observed effects should be considered preliminary evidence of the modulatory potential of CGL, requiring further investigation through mechanistic studies, toxicity evaluation, testing against a larger number of strains, and *in vivo* assays to better determine its therapeutic applicability.

Importantly, the statistically significant reductions in MIC observed for norfloxacin against *S. aureus* 10 and ciprofloxacin against *S. aureus* K2068 should be interpreted cautiously. Although the addition of CGL reduced the MIC values from 256 to 170.6 μg/mL for norfloxacin and from 32 to 13.3 μg/mL for ciprofloxacin, respectively, these reductions did not bring the isolates into the clinically susceptible range according to established interpretive criteria. Thus, the observed changes represent a measurable *in vitro* modulatory effect but do not indicate restoration of antibiotic susceptibility or establish clinical efficacy. The distinction between statistical significance and clinical relevance is particularly important in this context, as the magnitude of the MIC reduction was insufficient to overcome the high level of resistance exhibited by the tested isolates. Therefore, these findings should be considered preliminary and hypothesis-generating, supporting further investigation into the mechanisms underlying the interaction between CGL and quinolone antibiotics rather than immediate therapeutic application.

In some bacterial strains, CGL did not exhibit direct activity, nor did it enhance antibiotic activity, particularly in assays using Gram-negative strains. Nonetheless, previous studies have explored the antibacterial effect of lectins from the *Canavalia* genus (Santos et al., 2021). Jin et al. (2019) reported that *C. ensiformis* lectin (ConA) can prevent the initial adhesion of microbial cells to tissues, producing an anti-biofilm effect in enterohemorrhagic *E. coli* and *Listeria monocytogenes* strains. Other lectins, such as *C. villosa* lectin (Cvill), have shown antiproliferative activity through the induction of apoptosis in cancer cells, similar to what is observed with ConA (Lossio et al., 2023).

The observation that CGL was more effective in modulating resistance in *S. aureus* than in Gram-negative isolates is consistent with trends observed for other plant-derived antimicrobials. For instance, Oluyele (2025) demonstrated that the seed oil of Phoenix dactylifera (PDEO) exhibited lower MIC values and faster killing kinetics against Gram-positive bacteria, such as *S. aureus* and *S. pneumoniae*, compared to Gram-negative species like *K. pneumoniae*. This difference in susceptibility is often attributed to the complex outer membrane of Gram-negative bacteria, which acts as a barrier to many natural bioactive compounds.

The representativeness of the bacterial panel used in this study must be considered when interpreting the results. Although *S. aureus* and *E. coli* are among the most clinically significant pathogens associated with AMR (Antimicrobial resistance), our findings regarding the modulatory potential of CGL were largely confined to *S. aureus* quinolone-resistant models. The lack of significant activity against the tested Gram-negative clinical isolates suggests that CGL’s efficacy may be species-specific or dependent on particular cell wall compositions and resistance mechanisms. Therefore, these results should not be generalized to all multidrug-resistant bacteria at this stage.

Although several mechanisms have been proposed for lectins, such as membrane destabilization or interference with homeostasis, this study did not conduct direct mechanistic assays. To definitively prove that CGL inhibits the NorA or MepA pumps, future research must include fluorophore accumulation/efflux assays to measure transport kinetics, and RT-qPCR to determine if CGL affects the expression of the genes encoding these proteins.

The interaction with sugars alone is not the sole determinant for developing antimicrobial activity. According to Delatorre et al. (2007), CGL likely has the ability to bind and transport secondary metabolites, which could exert effective action against microorganisms. This supports the idea that CGL may facilitate the activity of these chemical compounds, as *C. gladiata* seeds contain various bioactive compounds with proven antimicrobial properties, such as terpenes, alkanes and alkenes, phenols, among others (More et al., 2022; Pal et al., 2022).

Previous studies have shown that substances such as CCCP and chlorpromazine are considered effective efflux pump blockers, which is why they are used as standard inhibitors of these proteins (Sousa Júnior et al., 2021; Oliveira-Tintino et al., 2023; Barbosa et al., 2023). Research on efflux pump inhibition by lectins, whether of animal or plant origin, is scarce, with most studies focusing only on direct activity (Murugesan and Gunasagaran, 2021; Arfin et al., 2022; Jeyachandran and Vibhute, 2024). Nonetheless, this does not diminish the importance of working with this type of natural product, as there are already published examples in the literature demonstrating significant antibacterial potential.

It should be noted that the present study did not directly quantify the expression levels of NorA and MepA. The evaluation was based on well-established efflux pump–carrying strains commonly used in modulation assays and on the response to known efflux pump inhibitors. Therefore, while the observed effects are consistent with a possible interaction with efflux-mediated resistance mechanisms, additional studies involving gene expression analyses and overexpression models would be necessary to confirm the specific involvement of these pumps.

Therefore, the findings obtained in this study should be interpreted cautiously. Although CGL exhibited limited direct antibacterial activity and only moderate modulatory effects, the results contribute to the growing body of evidence investigating lectins as potential adjuvants against multidrug-resistant bacteria. Additional studies are necessary to determine whether structural modifications, alternative experimental conditions, or combination approaches could enhance its biological activity. Furthermore, it is important to note that all microbiological assays were conducted in technical triplicate. While this approach ensures the precision of the reported MIC values for the tested instances, the lack of independent biological replicates performed on different days is a limitation of the present study.

## 5 Conclusion

The results obtained in this study indicate that CGL has weak intrinsic antibacterial activity against the tested bacterial strains, including multidrug-resistant isolates, with MIC values ≥1,024 μg/mL. However, when used at a sub-inhibitory concentration, CGL moderately enhanced the activity of selected quinolone antibiotics, reducing the MIC of norfloxacin against *Staphylococcus aureus* 10 and ciprofloxacin against *S. aureus* K2068. Nevertheless, these reductions were not sufficient to restore bacterial susceptibility according to clinically established therapeutic breakpoints. Therefore, the present findings support the potential of CGL as an antibiotic adjuvant or modulatory agent, rather than as a direct antibacterial agent. The mechanisms underlying these interactions remain unclear, and the present findings do not establish direct inhibition of the NorA or MepA efflux pumps by CGL. The responses observed in the presence of reference compounds such as CCCP should also be interpreted cautiously, as the growth-based assays do not distinguish efflux inhibition from broader physiological effects associated with proton motive force dissipation. Further studies involving direct measurements of substrate accumulation and efflux, appropriate assessments of bacterial viability, molecular characterization of efflux systems, toxicity evaluation, a broader panel of bacterial strains, and *in vivo* testing are necessary to clarify the mechanisms involved and determine the potential applicability of CGL in antibiotic combination strategies against multidrug-resistant bacteria.

## Data Availability

The raw data supporting the conclusions of this article will be made available by the authors, without undue reservation.

## References

[B1] Araújo L. D. S. Oliveira J. R. Silva L. N. Costa Júnior L. M. Silva M. D. P. Rocha C. Q. (2023). Prospecting plant extracts and bioactive molecules with antimicrobial activity in Brazilian biomes: a review. Antibiotics 12, 427. 10.3390/antibiotics12030427 36978294 PMC10044579

[B2] Araújo I. M. Pereira R. L. S. Araújo A. C. J. Gonçalves S. A. Tintino S. R. Oliveira-Tintino C. D. M. (2025). *In vitro* and *in silico* effect of Meldrum's acid-derived compounds on *Staphylococcus aureus* strains as NorA efflux pump inhibitors. Biophys. Chem. 316, 107344. 10.1016/j.bpc.2024.107344 39442379

[B3] Arfin N. Podder M. K. Kabir S. R. Asaduzzaman A. K. M. Hasan I. (2022). Antibacterial, antifungal and *in vivo* anticancer activities of chitin-binding lectins from tomato (*Solanum lycopersicum*) fruits. Arab. J. Chem. 15, 104001. 10.1016/j.arabjc.2022.104001

[B4] Arokiyaraj C. Tamilarasan K. Manikandan R. Janarthanan S. (2022). Purification and structural characterization of lectin with antibacterial and anticancer properties from grubs of hide beetle, *dermestes frischii* . Int. J. Biol. Macromol. 203, 312–332. 10.1016/j.ijbiomac.2022.01.099 35074334

[B5] Barbosa C. R. S. Macêdo N. S. Silveira Z. S. Rocha J. E. Freitas T. S. Muniz D. F. (2023). Evaluation of the antibacterial and inhibitory activity of the MepA efflux pump of *Staphylococcus aureus* by riparins I–IV. Arch. Biochem. Biophys. 748, 109782. 10.1016/j.abb.2023.109782 37839789

[B6] Bezerra G. A. Oliveira T. M. Moreno F. B. M. B. Rocha B. A. M. Delatorre P. Benevides R. G. (2007). Structural analysis of *Canavalia maritima* and *Canavalia gladiata* lectins complexed with different dimannosides: new insights into the understanding of the structure–biological activity relationship in legume lectins. J. Struct. Biol. 160, 168–176. 10.1016/j.jsb.2007.07.012 17881248

[B7] Clinical and Laboratory Standards Institute (CLSI) (2015). Performance Standards for Antimicrobial Susceptibility Testing (CLSI Document M100-S25). Wayne, PA: Clinical and Laboratory Standards Institute.

[B8] Costa A. R. Santos A. M. O. Barreto F. S. Costa P. M. S. Roma R. R. Rocha B. A. M. (2024). *In vitro* antiproliferative effects of *Vatairea macrocarpa* lectin on human tumor cell lines and *in vivo* toxicity in *Drosophila melanogaster* . Food Chem. Toxicol. 190, 114815. 10.1016/j.fct.2024.114815 38876381

[B9] Costa M. S. Silva A. R. P. Araújo N. J. S. Paulo C. L. R. Ribeiro P. R. V. Tavares J. F. (2024). UPLC–QTOF-MS/MS analysis of saponin-enriched fractions from *Calliandra umbellifera* and antibacterial activity against MDR bacteria. Phytochem. Lett. 59, 64–68. 10.1016/j.phytol.2023.11.002

[B10] Coutinho H. D. M. Costa J. G. M. Lima E. O. Falcão-Silva V. S. Siqueira-Júnior J. P. (2008). Enhancement of antibiotic activity against multiresistant *Escherichia coli* by *Mentha arvensis* and chlorpromazine. Chemotherapy 54, 328–330. 10.1159/000151267 18698137

[B11] Delatorre P. Rocha B. A. M. Souza E. P. Oliveira T. M. Bezerra G. A. Moreno F. B. M. B. (2007). Structure of a lectin from *Canavalia gladiata* seeds. BMC Struct. Biol. 7, 52. 10.1186/1472-6807-7-52 17683532 PMC1955443

[B12] El-Araby M. M. El-Shatoury E. H. Soliman M. M. Shaaban H. F. (2020). Characterization and antimicrobial activity of lectins from Egyptian leguminous seeds. Amb. Express 10, 1–14. 10.1186/s13568-020-01024-4 PMC7229064 32415415

[B13] Fonseca V. J. A. Braga A. L. Almeida R. S. Silva T. G. Silva J. C. P. Lima L. F. (2022a). Lectins ConA and ConM inhibit *Candida albicans* and *Candida tropicalis* . Arch. Microbiol. 204, 346. 10.1007/s00203-022-02959-x PMC9127036 35608680

[B14] Fonseca V. J. A. Braga A. L. Ribeiro Filho J. Teixeira C. S. Hora G. C. A. Morais-Braga M. F. B. (2022b). Antimicrobial properties of lectins: a review. Int. J. Biol. Macromol. 195, 163–178. 10.1016/j.ijbiomac.2021.11.209 34896466

[B15] Freitas T. S. Campina F. F. Costa M. S. Rocha J. E. Cruz F. P. Pinheiro J. C. A. (2021). Antibacterial activity of *Manilkara zapota* against MDR bacteria. Cell. Mol. Biol. 67, 116–124. 10.14715/cmb/2021.67.1.18 34817358

[B16] Houghton P. J. Howes M. J. Lee C. C. Steventon G. (2007). Uses and abuses of *in vitro* tests in ethnopharmacology: visualizing an elephant. J. Ethnopharmacol. 110 (3), 391–400. 10.1016/j.jep.2007.01.032 17317057

[B17] Jain M. Amera G. M. Muthukumaran J. Singh A. K. (2022). Biological role of plant defense proteins. Biocatal. Agric. Biotechnol. 40, 102293. 10.1016/j.bcab.2022.102293

[B18] Jeyachandran S. Vibhute P. (2024). Lectins as immunomodulatory and antibacterial proteins. J. Carbohydr. Chem. 43, 243–288. 10.1080/07328303.2024.2410792

[B19] Jin X. Lee Y. J. Hong S. H. (2019). *Canavalia ensiformis* lectin inhibits biofilm formation. J. Appl. Microbiol. 126, 300–310. 10.1111/jam.14108 30240117

[B20] Kumar V. Yasmeen N. Pandey A. Chaudhary A. A. Alawam A. S. Rudayni H. A. (2023). Antibiotic adjuvants: synergistic tool to combat multi-drug resistant pathogens. Front. Cell. Infect. Microbiol. 13, 1293633. 10.3389/fcimb.2023.1293633 38179424 PMC10765517

[B21] Laemmli U. K. (1970). Cleavage of structural proteins during the assembly of the head of bacteriophage T4. Nature 227, 680–685. 10.1038/227680a0 5432063

[B22] Li T. Wang Z. Guo J. Fuentes-Nunez C. Wang J. Han B. (2023). Bacterial resistance mechanisms and global health implications. Sci. Total Environ. 860, 160461. 10.1016/j.scitotenv.2022.160461 36435256 PMC11537282

[B23] Lossio C. F. Osterne V. J. S. Pinto-Junior V. R. Chen S. Oliveira M. V. Verduijn J. (2023). Structural characterization of an antiproliferative lectin. Int. J. Mol. Sci. 24, 15966. 10.3390/ijms242115966 37958949 PMC10649158

[B24] Matamoros-Recio A. Franco-Gonzalez J. F. Forgione R. E. Torres-Mozas A. Silipo A. Martín-Santamaría S. (2021). Computational explorations in bacterial membranes. ACS Omega 6, 6041–6054. 10.1021/acsomega.0c05590 33718695 PMC7948216

[B25] More K. Tayade S. Gawande P. Manik S. Shelke D. (2022). Antioxidant and antimicrobial potential of *Canavalia gladiata* . Indian J. Nat. Prod. Resour. 13, 163–169. Available online at: https://www.researchgate.net/profile/Kamlakar-More-2/publication/362862441_Antioxidaand_antimicrobial_potential_of_Canavalia_gladiata_Jacq_DC_leaves_and_seeds_GC-MS_based_metabolic_profiling/links/6304bb755eed5e4bd112ff04/Antioxidant-and-antimicrobial-potential-of-Canavalia-gladiata-Jacq-DC-leaves-and-seeds-GC-MS-based-metabolic-profiling.pdf.

[B26] Moreno F. B. M. B. Delatorre P. Freitas B. T. Rocha B. A. M. Souza E. P. Facó F. (2004). Crystallization and preliminary X-ray diffraction analysis of the lectin from *Canavalia gladiata* seeds. Acta Crystallogr. D. Biol. Crystallogr. 60, 1493–1495. 10.1107/S0907444904014489 15272187

[B27] Murugesan A. K. Gunasagaran K. S. (2021). Bioactive lectin from *Pleurotus flabellatus* . Anal. Biochem. 635, 114450. 10.1016/j.ab.2021.114450 34767809

[B28] Oliveira-Tintino C. D. M. Tintino S. R. Araújo A. C. J. Barbosa C. R. S. Freitas P. R. Araújo Neto J. B. (2023). Efflux pump inhibitors against *Staphylococcus aureus* . Molecules 28, 1819. 10.3390/molecules28041819 36838807 PMC9961278

[B29] Oluyele O. (2025). Antimicrobial efficacy and time-kill kinetics of phoenix dactylifera L. seed oil against multidrug resistant pathogens from cancer patients. Prospects Pharm. Sci. 23, 1–9. 10.56782/pps.336

[B30] Pal M. Pandey V. S. Dhawal A. Upadhyay P. Vartika A Ratanajay S. (2022). Pharmacological activities of *Canavalia gladiata* . J. Drug Deliv. Ther. 12, 208–216. 10.22270/jddt.v12i5-s.5729

[B31] Santos V. F. Araújo A. C. J. Freitas P. R. Silva A. L. P. Santos A. L. E. Rocha B. A. M. (2021). Gentamicin complexed with lectin against MDR strains. Microb. Pathog. 152, 104639. 10.1016/j.micpath.2020.104639 33238197

[B32] Santos M. H. C. Santos V. F. Freitas P. R. Silva R. R. S. Roma R. R. Santos A. L. E. (2023). Lectin enhances neomycin activity. Int. J. Biol. Macromol. 236, 123941. 10.1016/j.ijbiomac.2023.123941 36893486

[B33] Silva N. R. G. Araújo F. N. (2021). Antibacterial activity of plant lectins. Braz. Arch. Biol. Technol. 64, e21200631. 10.1590/1678-4324-2021200631

[B34] Silva A. R. P. A. R. P. Costa M. S. Araújo N. J. S. Alencar G. G. Ribeiro P. R. V. Tavares J. F. (2024). Saponin-enriched fraction from *Sarcomphalus joazeiro* . S. Afr. J. Bot. 165, 324–330. 10.1016/j.sajb.2024.01.007

[B35] Silva J. C. J. C. Silva L. R. O. Sousa Júnior D. L. Pereira R. L. S. Freitas T. S. Paulo C. L. R. (2024). Polyoxometalates against MDR bacteria. Results Chem. 7, 101466. 10.1016/j.rechem.2024.101466

[B36] Silva L. Donato I. A. Bezerra R. S. Santos H. S. Bandeira P. N. Nascimento M. T. R. (2024). Chalcone antibacterial activity against resistant *Staphylococcus aureus* . Fundam. Clin. Pharmacol. 38, 60–71. 10.1111/fcp.12938 37497790

[B37] Sousa Júnior D. L. Cordeiro P. P. M. Barbosa C. R. S. Muniz D. F. Silveira Z. S. Macêdo N. S. (2021). Isoeugenol as efflux pump inhibitor. Life Sci. 285, 119940. 10.1016/j.lfs.2021.119940 34508763

[B38] Tamilarasan K. Annapoorani A. Manikandan R. Janarthanan S. (2021). Galactose-specific lectin from *Odoiporus longicollis* . Int. J. Biol. Macromol. 183, 1119–1135. 10.1016/j.ijbiomac.2021.05.054 33974923

[B39] World Health Organization (2024). World Health Statistics 2024: Monitoring Health for the SDGs. Geneva: World Health Organization.

[B40] Zhai X. Wu G. Tao X. Yang S. Lv L. Zhu Y. (2023). Success stories of natural product-derived compounds from plants as multidrug resistance modulators in microorganisms. RSC Adv. 13, 7798–7817. 10.1039/D3RA00184A 36909750 PMC9994607

